# Ceramide on the road to insulin resistance and immunometabolic disorders in transition dairy cows: driver or passenger?

**DOI:** 10.3389/fimmu.2023.1321597

**Published:** 2024-01-11

**Authors:** Yuchao Zhao, Huiying Zhao, Liuxue Li, Shiqing Yu, Ming Liu, Linshu Jiang

**Affiliations:** Beijing Key Laboratory of Dairy Cow Nutrition, College of Animal Science and Technology, Beijing University of Agriculture, Beijing, China

**Keywords:** adipokines, ceramide, dairy cows, fatty liver disease, inflammation, immunometabolic homeostasis, oxidative stress

## Abstract

Dairy cows must undergo profound metabolic and endocrine adaptations during their transition period to meet the nutrient requirements of the developing fetus, parturition, and the onset of lactation. Insulin resistance in extrahepatic tissues is a critical component of homeorhetic adaptations in periparturient dairy cows. However, due to increased energy demands at calving that are not followed by a concomitant increase in dry matter intake, body stores are mobilized, and the risk of metabolic disorders dramatically increases. Sphingolipid ceramides involved in multiple vital biological processes, such as proliferation, differentiation, apoptosis, and inflammation. Three typical pathways generate ceramide, and many factors contribute to its production as part of the cell’s stress response. Based on lipidomic profiling, there has generally been an association between increased ceramide content and various disease outcomes in rodents. Emerging evidence shows that ceramides might play crucial roles in the adaptive metabolic alterations accompanying the initiation of lactation in dairy cows. A series of studies also revealed a negative association between circulating ceramides and systemic insulin sensitivity in dairy cows experiencing severe negative energy balance. Whether ceramide acts as a driver or passenger in the metabolic stress of periparturient dairy cows is an unknown but exciting topic. In the present review, we discuss the potential roles of ceramides in various metabolic dysfunctions and the impacts of their perturbations. We also discuss how this novel class of bioactive sphingolipids has drawn interest in extrahepatic tissue insulin resistance and immunometabolic disorders in transition dairy cows. We also discuss the possible use of ceramide as a new biomarker for predicting metabolic diseases in cows and highlight the remaining problems.

## 1 Introduction

The physiological adaptation of dairy cows to lactation has been extensively studied in recent years since its success is critical to the success of the entire lactation process. However, the transition to lactation remains the most crucial period in a dairy cow’s production cycle (1). Higher energy nutrient requirements for colostrum and milk production and lower feed intake drive cows to experience negative energy balance (NEB) (2). Consequently, dairy cows mobilize fat reserves from their bodies to compensate for the energy deficit (3). As a result of the mobilization of body fat, there is a shift in the ratio of lipogenic compounds to glucogenic compounds, increasing plasma metabolites such as nonesterified fatty acids (NEFA) and beta-hydroxybutyric acid (BHBA), which may increase the risk of metabolic diseases such as fatty liver disease (FLD) and ketosis (4, 5).

One of the critical homeorhetic adaptation strategies to successfully initiate lactogenesis and sustain galactopoiesis is the occurrence of insulin resistance in extrahepatic tissues and the decrease in pancreatic insulin secretion (6). A side effect of insulin insensitivity is the catabolism of fat depots and skeletal muscle, with the former often exacerbating the hepatocyte’s capacity to fully metabolize fatty acids (FA) and rendering cows more susceptible to fatty liver and ketosis (7). It was shown that elevated fatty acid (FA) availability promotes the accumulation of ceramide, a sphingolipid linked to the development of systemic insulin resistance (8). There is historical evidence that ceramide plays a role in downregulating cell proliferation, activating cell differentiation, and initiating programmed cell death mediated by caspases (9). Ceramides are now receiving attention for their contributions to the development of type 2 diabetes, non-alcoholic FLD (NAFLD), cardiovascular disease, and neurodegenerative disorders in humans. The role of ceramides in regulating physiological functions is well characterized, and the level of ceramides has been linked with metabolic syndromes, including FLD (10), obesity (11), and insulin resistance (12). Inhibition of ceramide production ameliorates metabolic disease characteristics, including insulin resistance, glucose intolerance, and diabetes in rodents (13).

In recent years, lipidomics research on the lipid composition of plasma or tissue in transition dairy cows has found correlations between sphingolipid profiles and physiological adaptations. Cows were shown to have changed sphingolipid levels near calving in their plasma, livers, adipose tissues, and skeletal muscles (8, 14, 15). Furthermore, comparisons between cows of different adiposity indicated a relationship between body fat mobilization and concentrations of ceramide, hexasylceramide, and lactosylceramide in plasma and liver, as well as between insulin resistance and specific ceramide, during the transition period (8, 16). Despite its potential role as a biomarker, a recent study also suggests that ceramide could drive the development of insulin sensitivity in primary bovine adipocytes (17). Therefore, understanding the possible roles of ceramides in modulating insulin signaling and immunometabolic disorders could reveal new nutritional strategies for preventing metabolic diseases in transition cows. In the present review, we will highlight the recent findings and some of the remaining gaps in understanding the role of ceramides in the immunometabolic health of periparturient cows.

## 2 Ceramide biosynthesis and metabolism

**Figure 1:**
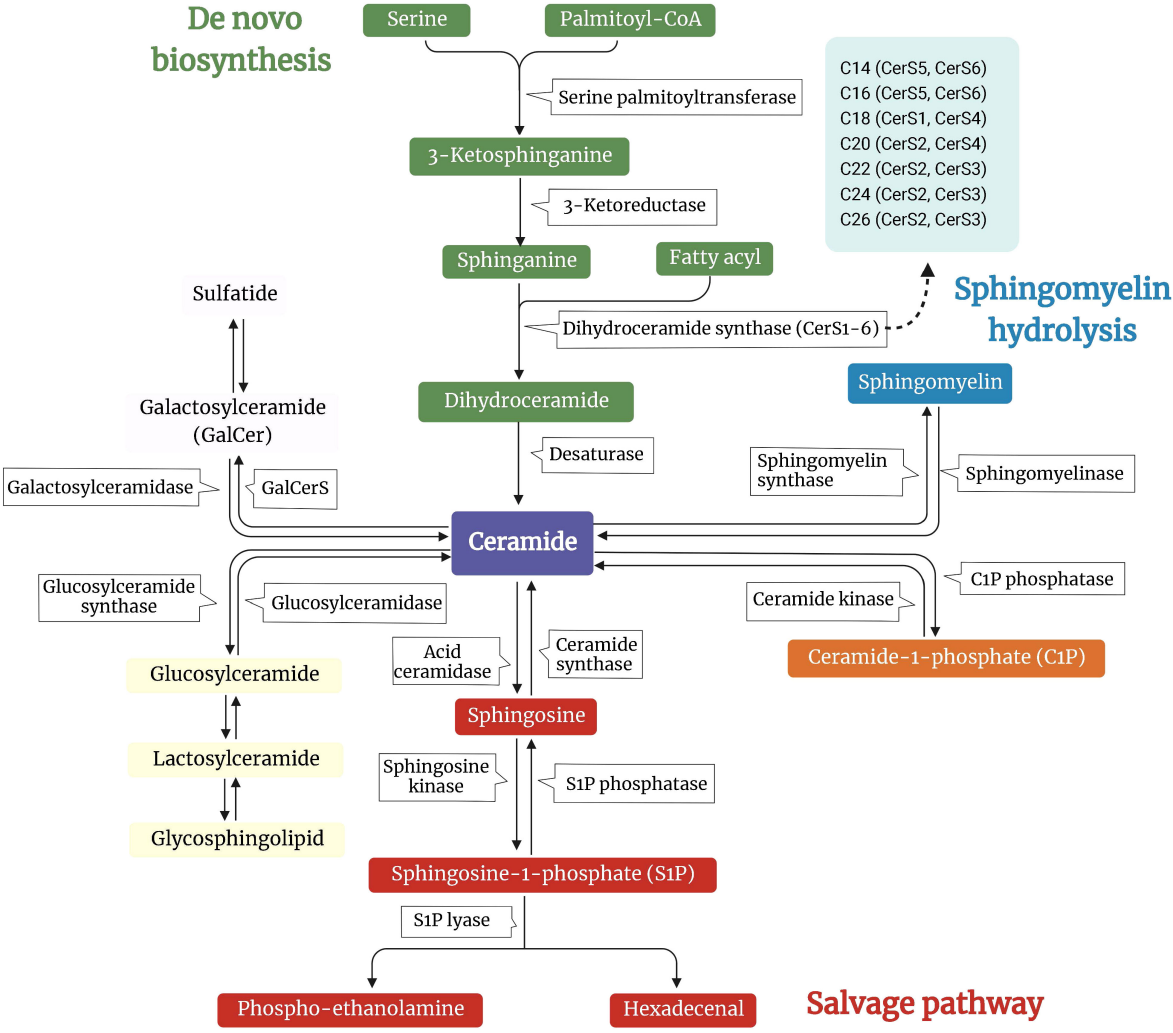
Overview of the metabolic pathways involved in the synthesis of ceramide. A schematic depiction of the major pathways controlling ceramide levels: *de novo* synthesis (green arm), sphingomyelin hydrolysis (blue arm), and salvage pathways (pink arm). In the *de novo* pathway, ceramide synthesis starts by condensing L-serine and palmitoyl-CoA to 3-ketosphinganine. This intermediate is reduced to sphinganine by 3-ketosphinganine reductase. Ceramide synthases (CerS) and the different isoforms (CerS1-6) are responsible for reactions that produce different species of ceramides. In the sphingomyelin hydrolysis pathway, sphingomyelin is hydrolyzed into ceramide by sphingomyelinase. For the salvage pathway, sphingomyelin is disassembled to ceramide by sphingomyelinase, which can further be metabolized to sphingosine by ceramidase. Ceramides serve as substrates for more complex sphingolipid species, such as glucosylceramides and galactosylceramides, which can be further modified.

Ceramides are structurally related molecules that form the core structure of the broader family of bioactive lipids found in all eukaryotes, sphingolipids (18). Sphingolipids (e.g., ceramides, sphingomyelins, sphingosine, sphingosine-1-phosphate (S1P), and gangliosides) are considerably less numerous than glycerolipids, constituting between 2% and 15% of the total cellular lipidome. Studies conducted in the middle of the 1980s identified the bioactive roles of sphingosine, ceramide, and S1P. They have profound biological functions despite being relatively insignificant membrane components, affecting the physiochemical characteristics of lipid bilayers and controlling the functioning of receptors and intracellular proteins (18). In mammals, ceramide can be produced by three primary pathways: *de novo* biosynthesis, hydrolysis of complex sphingolipids, and the salvage pathway (**Figure 1**).

### 2.1 *De novo* biosynthesis

The endoplasmic reticulum (ER) is responsible for the *de novo* synthesis of ceramides. A multistep enzymatic cascade begins with palmitoyl-CoA condensation with serine to form 3-ketosphinganine, a reaction catalyzed by serine palmitoyltransferase (SPT) (19). Serine palmitoyltransferase long-chain base subunits (SPTLC) 1 and 2 are crucial for enzyme activity. Other subunits, such as SPTLC3, change the substrate’s selectivity and enable the incorporation of other FA or amino acids to generate less abundant sphingolipid species (20, 21).

The six ceramide synthases (CerS1-6) catalyze the acylation of dihydrosphingosine to dihydroceramide. Despite carrying out the same chemical reaction, CerS isoforms differ in their specificity for the length of the acyl-CoA chain. For example, CerS5 and CerS S6 prefer C14:0 and C16:0, CerS S4 prefers C18:0 and C20:0, CerS3 prefers C22-26:0, CerS2 prefers C20-C26:0, and CerS1 prefers C18:0 (22). Members of the CerS family exhibit variable, tissue-specific expression patterns. In conjunction with the availability of various fatty acyl-CoA, it contributes to a complicated, tissue-specific distribution pattern of ceramides with varying acyl chain lengths (23). Subsequent desaturation yields the generation of ceramide from dihydroceramide. The last step in ceramide *de novo* biosynthesis requires dihydroceramide desaturases (DES1 and DES2), which add a crucial double bond into the sphingoid backbone of dihydroceramides to generate ceramide (24, 25).

### 2.2 Sphingomyelin hydrolysis

In the sphingomyelin (SM) hydrolysis pathway, sphingomyelinases (SMase) hydrolyze SM, releasing ceramides and phosphocholine (26). According to their preferred pH and subcellular location, SMases can be divided into three groups: acidic, neutral, and alkaline. The only organs in which alkaline SMase is expressed are the liver and the intestines, and it aids in the breakdown of SM from foods (26). However, neutral and acid SMases are widely expressed and critical regulators of SM degradation in most tissues (26).

### 2.3 Salvage pathway

In the salvage pathway of ceramide synthesis, also known as the recycling pathway, S1P, and sphingosine are converted to ceramide by S1P phosphatase and CerS. Ceramidases (CDases) degrade ceramide further into sphingosine (27). Based on the pH at which they function best, several CDases have been found and connected to other cellular compartments, such as SMase (acidic, neutral, and alkaline). While neutral or alkaline CDases have been recovered from nuclear membranes and mitochondria, acid CDases are lysosomal (28, 29). Sphingomyelin synthase transfers a phosphocholine group from phosphatidylcholine to ceramide, forming SM (30).

Ceramides are transferred from the ER to the Golgi apparatus through vesicular transport or the ceramide transport proteins. Ceramide is transformed to SM or glycosylceramide (including glucosylceramide and galactosylceramide) in the Golgi lumen. In the presence of sulfuric acid esters, galactosylceramide is transformed into sulfatides; glucosylceramide can be converted to lactosylceramide by adding galactose, which produces lactosides, gangliosides, and globosides (18). Sphingolipids are found in the Golgi membrane’s inner leaflet, making it easier for them to move to the outer leaflet as soon as the vesicles reach the plasma membrane (31).

### 2.4 Cellular stimuli-mediated ceramide generation

**Figure 2:**
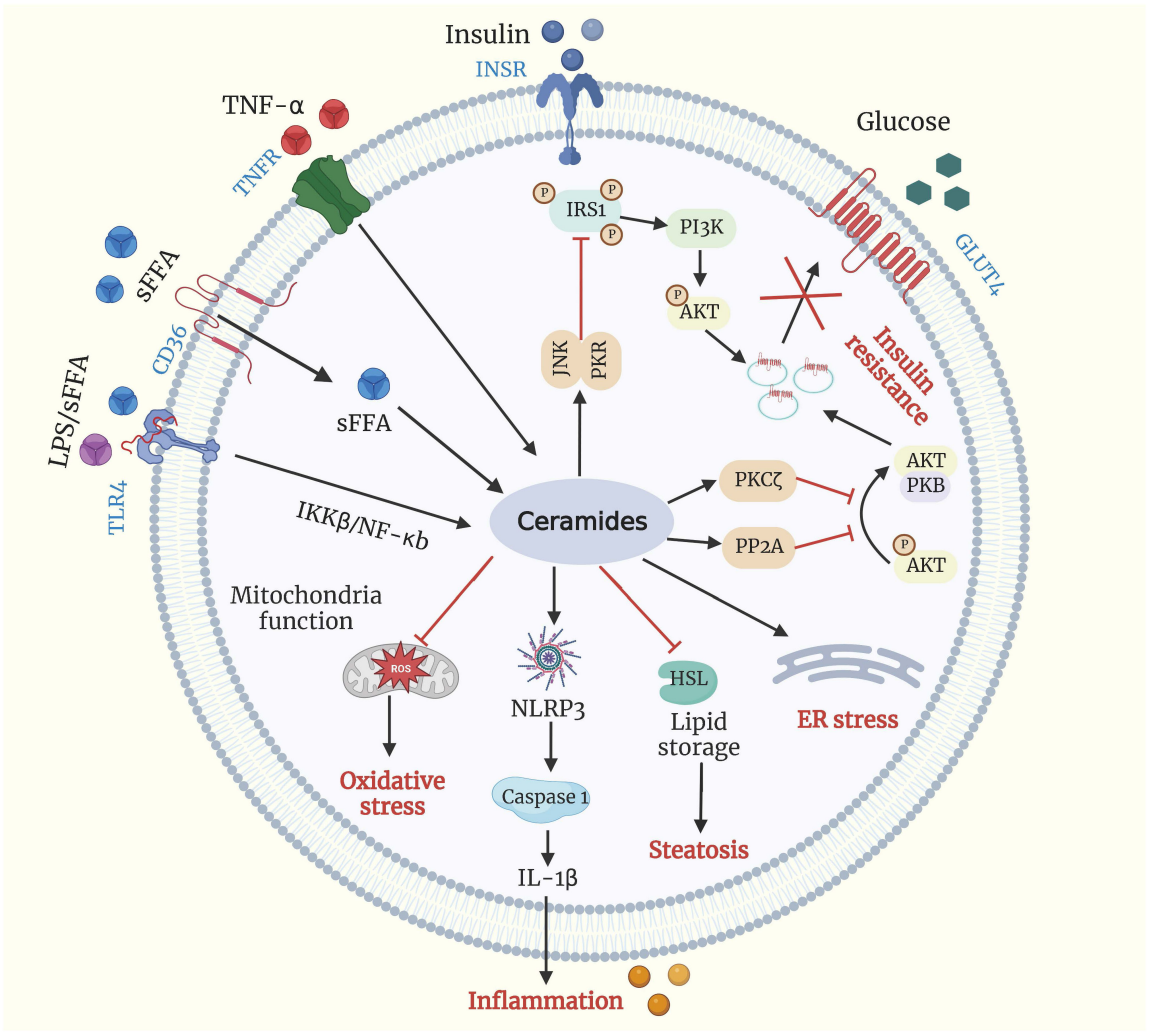
Ceramides and metabolic dysfunction. Ceramide production is driven by excessive influx of sFFA, LPS, and TNF-α. Ceramide can activate PKCζ, PP2A, and PKR/JNK, all of which can impair insulin signaling by blocking the translocation of (GLUT4) to plasma membrane. Ceramide accumulation promotes excessive lipid storage and steatosis by inhibiting HSL, inhibits mitochondrial capacity leading to oxidative stress, induces ER stress, and increments inflammation by activating the NLRP3 inflammasome. Akt/PKB, protein kinase B; CD-36, cluster of differentiation 36; ER, endoplasmic reticulum; GLUT4, glucose transporter 4; HSL, hormone-sensitive lipase; IL-1β interleukin 1β; INSR, insulin receptor; IRS1, insulin receptor substrates 1; LPS, lipopolysaccharide; NLRP3, NLR family pyrin domain containing 3; PI3K, phosphoinositide 3-kinase; PKCζ, protein kinase Cζ; PKR/JNK, double-stranded RNA-dependent protein kinase/c-Jun N-terminal kinase axis; PP2A, protein phosphatase 2A; sFFA, saturated free fatty acid; TLR4, Toll like receptor 4; TNF-α, tumor necrosis factor-α; TNFR, tumor necrosis factor-α receptor.

Both physiological and pathological changes influence cellular and circulatory ceramide concentrations. TNF-α can stimulate the production of ceramide (32) (**Figure 2**). TNF-α binds to its plasma membrane receptor, activating acid and neutral SMase (33). Acid SMase knock out mice are resistant to TNF-α-induced liver injury (34), and acid SMase activation contributes to TNF-α-induced hepatocyte apoptosis (34). Furthermore, a synergistic effect of saturated free fatty acids (sFFA) and lipopolysaccharide can further stimulate ceramide synthesis (35, 36). It is believed that TLR-4 activation by LPS or sFFA results in the accumulation of ceramides due to an elevated level of enzymes responsible for ceramide synthesis (37).

### 2.5 Can rumen-derived ceramide impact host sphingolipid metabolism?

Although significant advances have been made in our knowledge of ceramide metabolism in non-ruminants over the past two decades, much information still needs to be provided on ruminants. The majority of ceramides are also produced by gastrointestinal microbes of the phylum Bacteroidetes (38), including common genera (i.e., *Bacteroides* and *Prevotella*), which compose approximately 40%–50% of the rumen microbiome on average (39). These dominant members of the gastrointestinal microbiome possess the enzyme SPT, which catalyzes the initial step of *de novo* ceramide synthesis. The most abundant Bacteroidetes-derived sphingolipids are dihydroceramides, ceramide phosphoethanolamines, and ceramide phosphoinositol (40). Work with rodents has demonstrated that sphingolipids from the gut can be transferred to other organs. For example, a study by Johnson et al. (41) demonstrated that bacterial-derived sphingolipids could transfer to host epithelial tissue and the hepatic portal vein. Additionally, bacterial-derived sphingolipids suppress the *de novo* synthesis of sphingolipids, reducing certain ceramide species in the intestine and liver (40). *Bacteroides* and *Prevotella* species that produce sphingolipids are abundant in the rumen, providing an endogenous reservoir of bacterial lipids. The following areas require further research in dairy cows: first, a better understanding of the diversity of sphingolipids produced by different rumen bacteria; second, how these initial structures are metabolized; and third, how bacterial sphingolipids may serve as signaling molecules for the host and contribute to host metabolism.

## 3 Prepartum dietary energy affects ceramide metabolism in cows

McFadden and Rico (42) reviewed the effects of dietary palmitic acid on sphingolipid metabolism in dairy cows according to their group’s research. However, energy intake during the prepartum period can be an important factor affecting ceramide metabolism. Feeding a high-energy diet with a low-energy diet increases internal fat deposition. Based on adipose tissue transcriptomics, Moisá et al. (43) revealed that the KEGG pathway “sphingolipid metabolism” had a high impact with a slight activation in non-pregnant and non-lactating cows fed a high-energy diet. Feeding a high diet led to the down-regulation of mRNA expression of the gene N-acyl sphingosine amidohydrolase (acid ceramidase) 1 (*ASAH1*) and up-regulation of ceramide synthase 5 (*CERS5*) in adipose tissue (43). Using periparturient cows, Minuti et al. (44) observed that high-energy diets prepartum enhanced the mRNA abundance in adipose tissue of delta 4-desaturase, sphingolipid 1 (*DEGS1*) and *SPTLC2* (at −14 d from parturition), which are essential genes coding key enzymes in ceramide *de novo* biosynthesis. It was shown that more than half of ceramide species in the liver were up-regulated in dairy cows fed a prepartal high-energy diet at −8 d from parturition (45). However, Qin et al. (45) observed no differential gene expression related to ceramide metabolism. These studies suggest that feeding high-energy diets may generate divergent results on ceramide metabolism in dairy cows, and multiomics studies of liver and adipose tissue are warranted.

## 4 Ceramides and insulin resistance

### 4.1 Mechanisms found in non-ruminants

Insulin resistance can manifest as a reduction in insulin sensitivity (the insulin concentration that induces a half-maximal response), a reduction in insulin responsiveness (the maximal effect of insulin on insulin-sensitive tissues), or both simultaneously (46). There is evidence that higher plasma ceramide levels are associated with insulin resistance and the development of metabolic syndrome (47). Increased palmitoyl-CoA availability increases *de novo* ceramide synthesis and sphingomyelin hydrolysis in peripheral tissues of obese insulin-resistant animals with hepatic steatosis (37, 48). Haus et al. (49) reported that circulating ceramide levels are directly linked to insulin resistance in obese individuals with type 2 diabetes. They discovered that insulin sensitivity is negatively associated with the ceramide species C18:0, C20:0, and C24:1, as well as the overall plasma ceramide level in such patients. Chavez and Summers (50) also highlighted the significance of ceramides in insulin resistance and offered a new hypothesis implicating ceramides as a crucial agent in establishing insulin resistance. In addition, pharmacological inhibition of ceramide biosynthesis has been proven to promote glucose metabolism and insulin sensitivity (47). CerS inhibition by fenretinide prevents lipid-induced insulin resistance (51). Additionally, Watson et al. (52) found that upregulating *de novo* synthesis via SPT modulation increased serum ceramide content. In contrast, selective inhibition with myriocin decreased ceramide levels, alleviated obesity-derived atherosclerosis (53), enhanced insulin sensitivity (13), and decreased body weight (52, 54).

Ceramides play essential roles in insulin resistance due to their ability to inhibit insulin-stimulated glucose transport (**Figure 2**). Ceramide’s ability to inhibit the Akt/protein kinase B (PKB) pathway, a serine/threonine kinase required for insulin signaling and insulin-mediated stimulation of glucose metabolism, is the basis for this action. As a result, glucose transporter 4 (GLUT4) translocation, glucose uptake, and glycogen synthesis are all inhibited (54, 55). In addition, ceramides have also been demonstrated to disrupt glucose metabolism by acting on Akt/PKB signaling pathways via two distinct signaling molecules: protein phosphatase 2A (PP2A) and protein kinase C-zeta (PKCζ) (56, 57).

The phosphorylation of insulin receptor substrate-1 (IRS1) has also been proposed as a mechanism for ceramide-induced insulin-antagonistic effects. IRS1 regulates the binding of insulin and insulin-like growth factor 1 to intracellular receptors, thereby activating downstream signaling pathways (58). In addition, Yu et al. (59) reported that ceramides are directly involved in the phosphorylation of IRS1 and may negatively influence insulin signaling. Ceramides block IRS1 by triggering the protein kinase-mediated double-stranded RNA-dependent protein kinase/c-Jun N-terminal kinase axis (PKR/JNK), leading to the inhibition of insulin receptor signaling (60).

### 4.2 Associations observed in transition dairy cows

**Figure 3:**
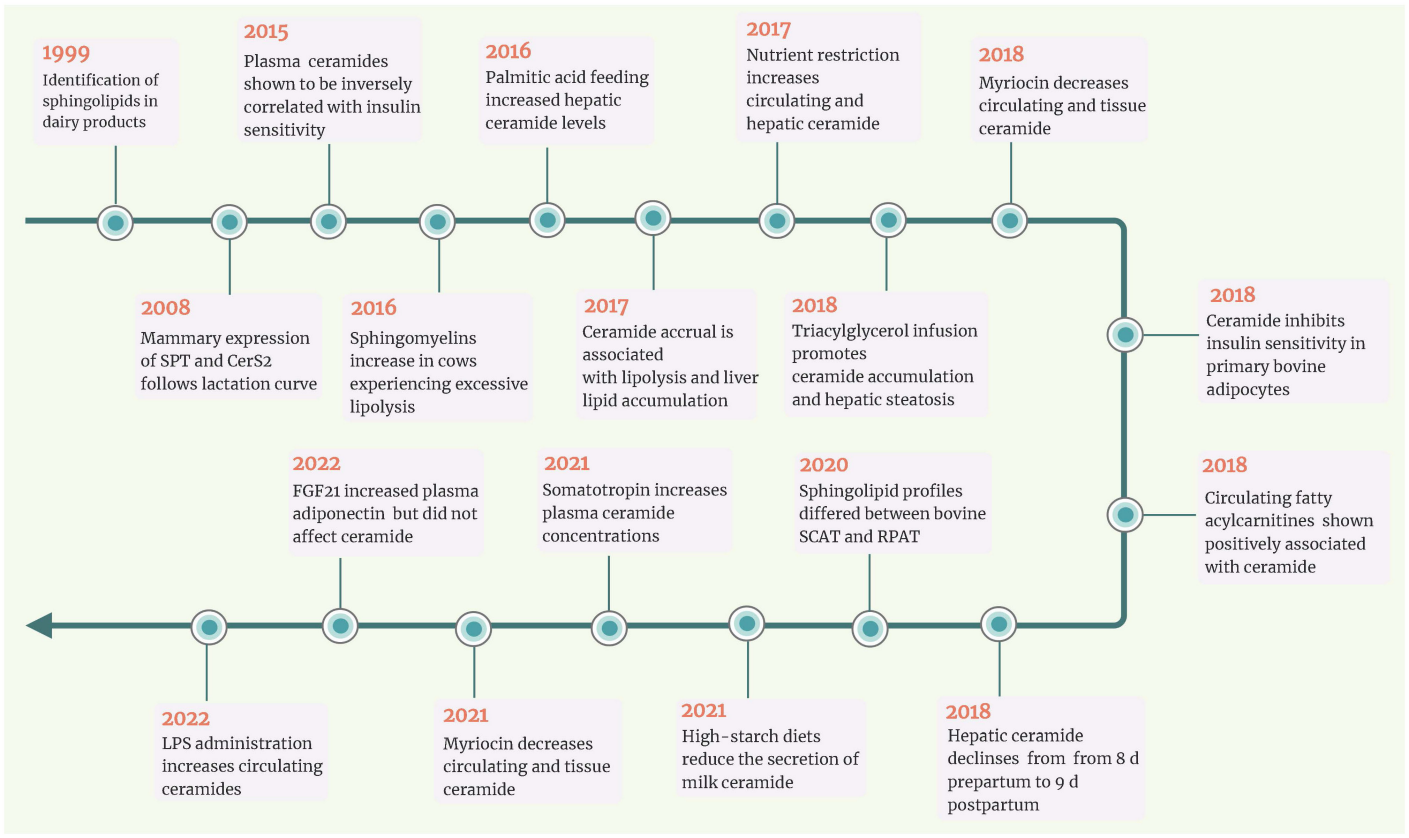
Timeline of key events for ceramide in ruminants. In the last decade, a series of studies revealed that circulating and/or hepatic ceramide levels were negatively associated with insulin sensitivity in transition dairy cows. Simultaneously, nutrient restriction and lipid infusion both increased ceramide accumulation in non-lactating dairy cows. Research in bovine adipocytes and non-lactating ewes has revealed that myriocin could inhibit ceramide *de novo* synthesis. CerS2, ceramide synthases 2; FGF21, fibroblast growth factor 21; LPS, lipopolysaccharide; RPAT, retroperitoneal adipose tissue; SCAT, subcutaneous adipose tissues; SPT, serine palmitoyltransferase.

Insulin resistance in periparturient cows prioritizes glucogenic resources for critical activities, fetal growth, and milk production and enhances the mobilization of FA from adipose tissue (7). Although growth hormone has a role in insulin resistance in transition cows (7), there is strong evidence that long-chain sFFA operates as an antagonist of systemic insulin sensitivity (61, 62). The ability of excess saturated fatty acyl-CoA to reduce insulin sensitivity appears to be regulated by the structurally diverse sphingolipid ceramide, according to biomedical research identifying ceramide as the agent responsible for insulin resistance in obese and diabetic non-ruminants with excess sFFA. Scientists have been able to study sphingolipids in large tissue biobanks thanks to advances in lipidomics. Crucial links between circulating ceramides, lipolysis, and insulin sensitivity have been shown. Ceramide’s involvement in aiding the development of extrahepatic insulin resistance in cows moving from gestation to lactation was also examined (**Figure 3**).

Compared with cows fed a controlled-energy diet, prepartal high-energy diet was reported to increase the levels of ceramides in adipose tissue and SM in the liver (15). Rico et al. (16) revealed that circulating concentrations of total ceramide and monohexosylceramide increased as lactation approached and that saturated ceramide and monohexosylceramide were higher in cows with higher adiposity (body condition score (BCS) >4.0) than in those with a lean phenotype (BCS<3.0). In addition, circulating ceramides (e.g., C24:0-ceramide) were positively associated with plasma NEFA and negatively related to systemic insulin sensitivity (measured with the revised quantitative insulin sensitivity check index (RQUICKI)) (16). Rico et al. (16) first demonstrated a modified plasma sphingolipidome in dairy cattle during the transition period, marked by an enhancement in plasma ceramides concurrent with the development of systemic insulin resistance. These results also supported the expected substrate-product relationship that drives *de novo* ceramide synthesis (63), whereby lipolytic-derived palmitic acid is utilized by SPT to form 3-ketosphinganine or elongated by elongases for utilization by CerS to form ceramide.

Given that indirect indices to estimate systemic insulin sensitivity in ruminants must be interpreted cautiously, there is a need for direct measurements of systemic insulin sensitivity that can be utilized to assess its association with sphingolipids throughout the transition period. Rico et al. (8) corroborated the inverse relationships between direct measures of systemic insulin sensitivity (employing intravenous insulin and glucose challenges) and peripartal contents of ceramide and glycosylated ceramide. According to Rico et al. (8), plasma sphingomyelin, a potential source of ceramides, reaches a minimum at parturition and is directly related to feed intake. However, Qin et al. (45) found that the hepatic content of most ceramide and sphingomyelin subtypes decreased in dairy cows from 8 days prepartum to 9 days postpartum. This may be due to enhanced lipoprotein export of ceramide when more FA reaches the liver (63), as indicated by the negative connection between postpartum plasma FA levels and hepatic ceramide contents (45).

### 4.3 What can we learn from non-ruminants?

Causality and correlation are incorrectly interchanged when an observational relationship between two events is considered inevitable rather than coincidental. Currently, the relationships between ceramide and insulin resistance in dairy cows are primarily based on associations, not cause-and-effect relationships garnered from controlled and intervening experimentation. In type 2 diabetic non-ruminant models, sustained hyperlipidemia shifts FA processing toward the hepatic synthesis and lipoprotein secretion of sphingolipid ceramide (63). In a study by Pires et al. (61), the authors show that intravenous infusion of tallow emulsion induces insulin resistance in Holstein cows by impairing insulin sensitivity and maximum responsiveness. Rico et al. (64) observed a delayed decline in plasma ceramide concentrations with the advancement of lactation during palmitic acid feeding. Increased plasma ceramide is associated with elevated basal NEFA, reduced adipose tissue NEFA disappearance to a glucose challenge, and milk yield (64). Davis et al. (65) first evaluated the link between controlled dietary restriction, ceramide levels, and systemic insulin sensitivity in nonpregnant and non-lactating cows. Nutrient restriction enhances ceramide accumulation in the liver, a response that is favorably related to the circulating ceramide supply (65). These controlled trials showed that ceramides might be intrinsically involved in homeorhetic adaptation to lactation.

However, whether and how ceramide induces insulin resistance remains unknown in dairy cows. It is well known that FA can also impair the insulin sensitivity of skeletal muscle and adipose tissue. Considering the positive correlation between circulating FA and ceramide, it is difficult to demonstrate whether the process of FA affecting insulin signal transduction depends on ceramide. Based on the research protocols and mechanisms in non-ruminant animals, Rico et al. (17) found that treating bovine adipocytes with C2:0-ceramide for 2 h decreased the ratio of pAkt to Akt and 2-deoxy-D-(3H)-glucose uptake (2DOG). In contrast, myriocin treatment decreased all ceramide species, increased the transport of 2DOG into cells, and enhanced insulin sensitivity (17). Myriocin is commonly used to test the hypothesis that ceramides diminish insulin sensitivity in rodent models. However, due to its high price and unknown risks to cow health, using myriocin for controlled trials in cows is not practical. Davis et al. (66) revealed that nutrition restriction elevated plasma C16:0-ceramide in non-lactating crossbred ewes and that myriocin inhibited this effect. Unfortunately, Davis et al. (67) did not report changes in insulin sensitivity.

From the above, it is evident that large *in vivo* and *in vitro* controlled studies are needed to validate the role of ceramides in insulin resistance in dairy cows. Using short ceramide species, such as C2 and C6, was common practice for cell-culture experiments because aqueous solutions made the administration of the ceramides easier. Nevertheless, among physiologically present ceramides, the length of the acyl chain plays an important role and is mainly responsible for determining the biological effects. Under physiological conditions, using short, nonphysiological ceramides in experimental settings may produce nonreproducible or irrelevant results. Consequently, there are still many unanswered concerns, such as the specific functions of different chain-length ceramides and whether the functions of ceramides are consistent in various organ tissues of dairy cows.

## 5 Might ceramide play a role in immunometabolic disorders of transition cows?

Studies in non-ruminants suggest that ceramides are causal agents of disease. In cardiac tissue biopsies, ceramides were higher in failing hearts and decreased in patients receiving a left ventricular assist device (68). In the liver, ceramide species were raised in patients with steatosis (69). In adipose tissues, ceramides were increased in patients with obesity (70) and diabetes (71). However, the roles of ceramides in critical metabolic organs, such as the adipose and liver, have not been widely characterized in cows. Considering the accrual of ceramide from late pregnancy to calving, it is possible that ceramides may be involved in some metabolic disorders (**Figure 2**). Observations in non-ruminants warrant further exploration in cows, which have significant implications for cows’ health.

### 5.1 Ceramide and hepatic steatosis

Fatty liver is a common disease in modern dairy farms, occurring to varying degrees during the periparturient period. A high influx of FA from adipose tissue lipolysis into the liver results in excessive triacylglycerol accumulation. In non-ruminants, the accumulation of triglycerides and ceramides in the liver leads to steatosis when the net influx of FA exceeds the excretion and oxidation of triglycerides (72). Ceramide production in NAFLD is predominantly attributed to activating the *de novo* synthesis pathway of ceramides in hepatocytes (73), evidenced by increased expression of SPT, CerS1, CerS2, CerS4, and CerS6 in the liver (74, 75). Steatosis is the initial stage of FLD, characterized by a steady rise in liver lipids (76). In dairy cows, Rico et al. (77) observed that intravenous triacylglycerol infusion increased the hepatic mRNA expression of *CERS2*, promoting ceramide accumulation and hepatic steatosis. Multiple recognized mechanisms lead to steatosis in dairy cows: greater absorption of FA, reduced secretion of FA, and suppressed FA oxidation (78).

The FA transporter proteins (FATP), FATP2, FATP5, and FA translocase CD36 are responsible for FA absorption in hepatocytes (79). Ceramides promote plasma membrane localization of CD36, which binds and transports FA into hepatocytes (80). Accordingly, in mice, reducing hepatic ceramides through the downregulation of DES1, a critical component of the ceramide *de novo* production pathway, reduced the expression of CD36, FATP2, and FATP5 and slowed FA uptake in the liver (81). Additionally, the liver exports triglycerides as very low-density lipoprotein (VLDL). It has been shown that inhibiting VLDL secretion increases the accumulation of triglycerides in the liver, resulting in steatosis (82). Increased VLDL export alleviated hepatic steatosis. Correnti et al. (83) employed a hepatic-specific acid ceramidase overexpression model to reduce hepatic ceramides in mice. The results showed that reducing hepatic ceramides helped relieve steatosis by increasing the release of VLDL. This suggests that hepatic ceramide inhibition can help prevent FLD.

Currently, liver lipidomic data related to hepatic steatosis in dairy cows are still lacking. The development of postpartum fatty liver is most frequently observed in cows that are overconditioned at the time of parturition. As reported by Pascottini (84), high basal lipolysis at the end of pregnancy enhanced the liver lipid level in overconditioned cows, but gene expression patterns related to sphingolipid metabolism did not change. In addition, insulin resistance is a cause and consequence of hepatic steatosis in non-ruminants (85). However, high-yielding dairy cows might not suffer from hepatic insulin resistance (86). Therefore, the role of ceramide in hepatic steatosis in dairy cows may differ from findings observed in rodent models, and further investigation is needed.

### 5.2 Ceramide and adipose tissue inflammation

Even without signs of microbial infection or other pathology, postpartum cows have been reported to exhibit a pronounced inflammatory response related to the end of pregnancy and the onset of lactation (87). A hallmark of an inflammatory state is the release of proinflammatory cytokines, which can result in various metabolic changes, such as anorexia, lipid mobilization, diminished insulin sensitivity, and decreased milk yield (88). For example, a recent study demonstrated that TNF-α can promote apoptosis, impair insulin sensitivity, and induces a lipolytic response in bovine adipocytes (89). In addition to playing a role in lipid storage and release, the metabolism of adipose tissue actively regulates homeostasis and the inflammatory response, making it crucial for studying sphingolipid biology (90). Intense adipose tissue remodeling accompanied by increased expression of an inflammatory phenotype by adipose tissue macrophages may impair the metabolic function of adipose tissue in dairy cows due to physiological changes associated with parturition, the onset of lactation, or prolonged periods of lipolysis (or both) (91).

**Figure 4:**
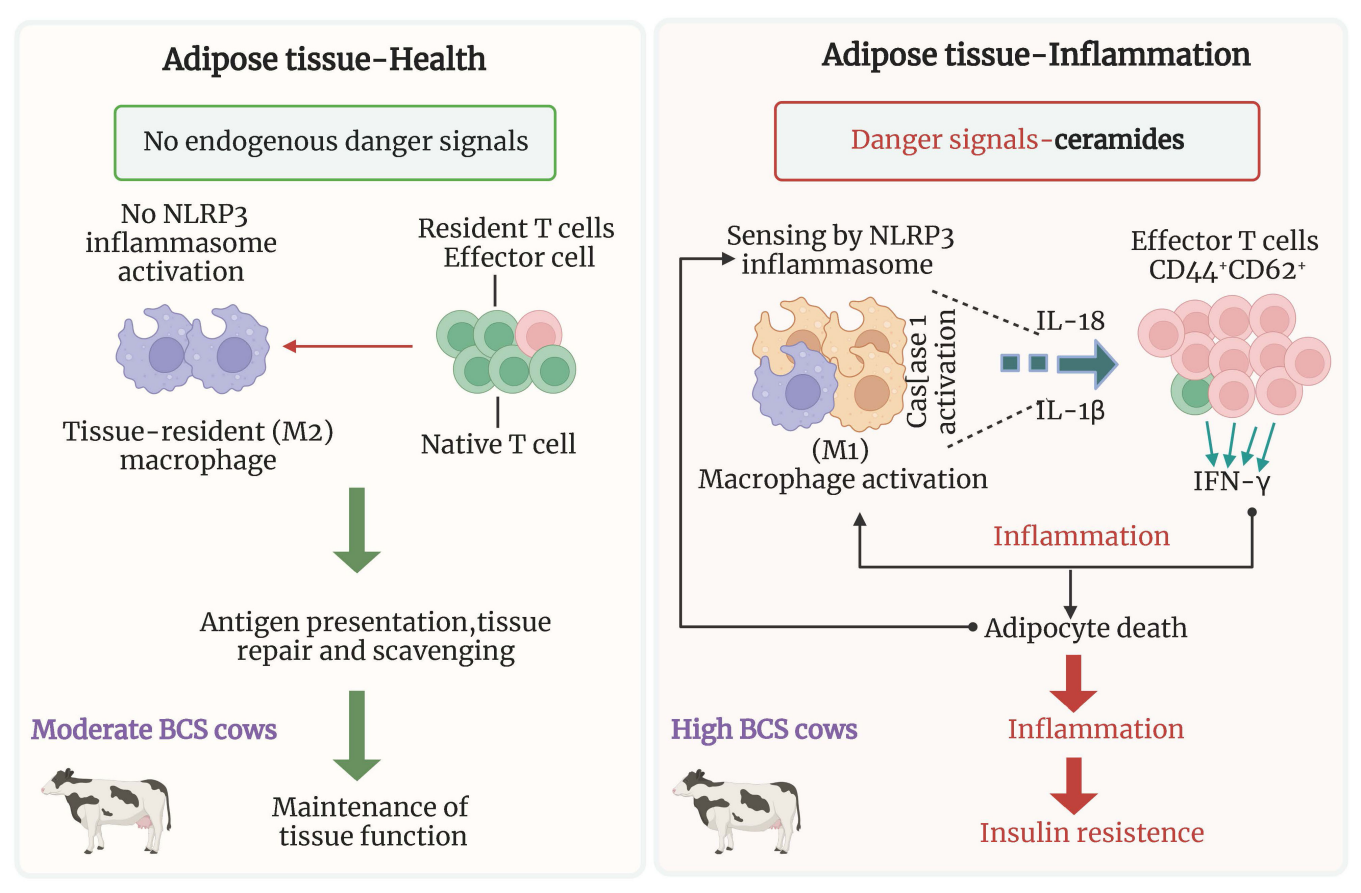
Hypothetical model of NLRP3 inflammasome activation in obese dairy cows. In the absence of danger signals in the normal state, tissue-resident macrophages and T cells may participate in the maintenance of adipose tissue function. In obesity, the NLRP3 inflammasome senses the obesity-associated danger signals such as ceramides leading to caspase-1 autoactivation and IL-1β and IL-18 production from ATM. Secondary signals from activated ATM to effector adipose T cells (defined as CD44^+^CD62^−^) sustain the reciprocal proinflammatory feed-forward cascade during obesity, leading to insulin resistance. ATM, adipose tissue macrophages; NLRP3, NLR family pyrin domain containing 3.

It is becoming increasingly apparent that sphingolipids can be intimately involved in inflammation (23). On the one hand, inflammatory cytokines such as TNF-α in serum and adipose tissue stimulate ceramide production via TLR4 and TNF-α dependent pathways. On the other hand, it was recently discovered that sFFA triggers inflammasome activation in macrophages, suggesting that lipotoxic intermediates such as ceramides may be responsible for inflammasome activation (92). Vandanmagsar et al. (93) confirmed this hypothesis in mice, demonstrating that ceramide increases active caspase-1 in macrophages and adipose tissue in an NLR family pyrin domain containing 3 (NLRP3)-dependent manner. It is widely known that various non-microbial danger-associated molecular patterns trigger the NLR family member NLRP3 inflammasome and promote inflammation by boosting the release of IL-1 and IL-18 (**Figure 4**). Therefore, Vandanmagsar et al. (93) first confirmed that ceramides themselves might trigger inflammation, enabling a positive feedback mechanism that would aggravate tissue damage.

Although this ceramide-NLRP3 inflammasome interaction may contribute to adipose inflammation, which contributes to insulin resistance in rodent and human adipocytes, direct evidence regarding the role of sphingolipids in bovine adipose or their ability to regulate NLRP3 and inflammation is lacking. The complex mechanisms lead to dysregulated inflammation and lipolysis in dairy cow’ adipose tissue (94). It should be noted that neutral lipids (such di- and monoglycerides), phospholipids (such as ceramides and sphingolipids), and oxylipids are inflammatory mediators in AT that come from lipids (94). More studies are required to define the role of ceramide in adipose tissue inflammation.

Furthermore, the adipose tissue of transition dairy cows from different sites (i.e., retroperitoneal adipose tissue (RPAT) and subcutaneous adipose tissue (SCAT)) differs in their metabolic adaptations (95). Leung et al. (96) found that the sphingolipid profiles differ between bovine RPAT and SCAT; in particular, the content of ceramides was higher in RPAT than in SCAT. These findings indicate that the pathways of sphingolipid metabolism, including *de novo* synthesis of ceramide, are also activated differently in RPAT and SCAT, which may explain their tissue-specific effects on insulin sensitivity and proinflammatory responses.

### 5.3 Ceramide and oxidative stress in liver and adipose tissue

Hepatic FA oxidation regulation primarily depends on mitochondria, the major organelle responsible for ATP production (97). Despite the hepatic ability to process FA, excess FA entering the liver causes lipotoxicity and impairs liver mitochondrial function (98). Cells with high levels of FA are more likely to produce reactive oxygen species (ROS), which can damage mitochondria and aggravate hepatic steatosis (99). In mature dairy cows with fatty liver, there was a notable decrease in the presence of mitophagy markers alongside an excess production of ROS (100).

As a derivative of FA, ceramide cannot be ignored. Mitochondrial membranes contain ceramides as physiological components. The interrelationship between ceramide and oxidative stress is well-known in non-ruminants (101). On the one hand, even though ceramides are essential for the normal function of mitochondria, excessive levels of mitochondrial ceramides can lead to mitochondrial dysfunction and impair respiratory capacity by generating ROS and causing increased oxidative stress, reducing ATP, disrupting the electron transport chain, causing apoptosis, and altering the permeability of the mitochondrial outer membrane (102).. On the other hand, H_2_O_2_, an inflammatory oxidant, and hypoxia, which causes irreversible impairment in multiple cellular systems, have been used to indicate that oxidant-induced apoptosis is mediated by the SM-ceramide pathway (103, 104). Indeed, if ROS can stimulate ceramide production, ceramide may inhibit isolated mitochondrial electron transport at complex III, increasing reactive oxygen species (105). Thus, it is possible that the coupling between oxidative stress and ceramide production is bidirectional.

Furthermore, the rapid expansion of adipocytes and adipose tissue increases intercapillary distance, reducing blood flow and oxygen delivery (106). Metabolic stress is accompanied by increased oxidative stress in cows with a BCS >3.5 before calving and greater BCS loss after calving (107). Oxidative stress diminishes the insulin response of adipocytes. It causes a vicious cycle in which oxidative stress exacerbates adipose tissue lipolysis and inflammation, facilitating further oxidative stress (93). In a study that provided nonpregnant, nonlactating cows with a ration of increasing energy density for 15 weeks to increase BW and body condition, the number of HIF-1α–positive cells in adipose tissue increased 3.3-fold from week 0 to 15, and HIF-1α protein levels increased 2.6-fold from week 0 to 8. The HIF-α family (HIF-1α, HIF-2α, and HIF-3α) is stabilized and activated under hypoxic conditions (108). In rodent models, activation of HIF-2α induced intestinal hypoxia and elevated ceramide levels led to NAFLD (109). The disruption of adipocyte HIF-1α ameliorates atherosclerosis by inhibiting ceramide production (110). Therefore, if the role of ceramide in the development of oxidative stress can be clarified in future studies, it may be possible to alleviate the vicious cycle of oxidative stress-inflammation-excessive lipolysis in overconditioned dairy cows’ adipose tissue by regulating the H1F-ceramide pathway.

## 6 Are ceramides predictive markers of transition cows’ health?

Elevated prepartum and postpartum NEFA concentrations as well as postpartum BHBA concentrations have been associated with a higher risk of abomasal displacement, hyperketonemia, retained placenta, metritis, and culling (111–113). Threshold values of NEFA and BHBA concentrations have been proposed for identifying animals at greater risk of the development of the aforementioned diseases. However, these values have not always been the same between studies and within diseases of interest (112–114). A longitudinal cows cohort study revealed a relationship between serum NEFA concentrations in the 14-d period before calving and the subsequent development of diseases (abomasal displacement, hyperketonemia, retained placenta, metritis), and optimal thresholds associated with postpartum diseases were between ≥260 and 300 µmol/L (115). However, considering the low accuracy, their use at the cow level should be performed with caution. Other studies have investigated the use of various lipid biomarkers, such as sphingomyelins, for the prediction of peripartal diseases (116). However, sample size limits prevent extrapolation and generalization.

In recent years, ceramides have garnered growing attention due to their regulation in numerous human diseases, including cancer, diabetes, multiple sclerosis, and coronary artery disease. In addition to being potential therapeutic targets, ceramides are increasingly considered intriguing diagnostic targets (117). As evidence from human studies indicates, the identification of ceramide as a strong predictor for disease would likely trigger the development of nutritional or pharmacological therapies that modulate ceramide synthesis as a preventive measure to enhance insulin sensitivity, control lipolysis, and support health and longevity. However, the efficacy of ceramides as predictors for disease relative to NEFA and BHBA has yet to be studied in dairy cows. McFadden group demonstrated that circulating C24:0-ceramide increased concurrently with lipolysis intensity (8, 16). Cows with significant fatty deposition in the liver also accumulated hepatic C24:0-ceramide (8), and liver and plasma ceramide patterns are comparable (8, 65). Compared with liver biopsy assessment, plasma ceramide evaluation is more rapid, timely, and convenient, although the liver is a major contributor to circulating sphingolipids (42). According to Humer et al. (116), more than half of the identified SM based on targeted metabolomics were elevated in cows with excessive lipolysis and diminished insulin sensitivity. Humer et al. (118) suggested that these novel biomarkers may be beneficial in practice for identifying high-risk cows sooner and treating or preventing the onset of illnesses linked to excessive lipid mobilization in cows.

However, judging biomarkers based on BCS or lipolysis degree is not enough. More comparisons in sphingolipid profiles between healthy and diseased cows still need to be made. Wu et al. (119) did not observe differences in the mRNA abundance of ceramide metabolism-related genes in the blood of cows with clinical ketosis vs. healthy cows by the RNA-Seq approach. Shahzad et al. (120) showed more extensive changes in the liver transcriptome than metabolome (−10 d relative to parturition) in cows overfed energy during the prepartum period and developing ketosis postpartum. Compared to cows that did not develop clinical ketosis, cows with clinical ketosis had higher mRNA abundance of *CERS2* and lower abundances of *SMPD3* and *CERS4* (120). Unfortunately, this study did not detect the levels of ceramide species, which may be due to the limitations of metabolomics in identifying lipids. Using untargeted lipidomics, we recently identified 26 ceramide species in the serum of cows in early-lactation (10-30 DIM). Our unpublished results showed that subclinical ketosis cows (n=8) had greater plasma concentrations of Cer(d16:0/16:0), Cer(d18:1/24:0), Cer(d18:1/16:0), and Cer(t18:0/24:0) than healthy cows (n=8). Furthermore, we employed targeted lipidomics to characterize the ceramide profiles in adipose tissue of healthy and subclinical ketosis cows. Only nine ceramide species were identified, which is subject to the limitations of standard availability. Compared with healthy cows, the concatenations of Cer(d18:1/24:0) and Cer(d18:1/16:0) were up-regulated in subclinical ketosis cows (unpublished data). However, the sample size was too small for candidate biomarker discovery.

Due to their ubiquitous nature and constant modifications during pathophysiology, it is implausible that a single ceramide could be used as a biomarker for a specific disease. To assess and understand which species and ratios could function as reliable biomarkers in the context of various pathophysiological metabolic status in cows, it is currently necessary to measure the complex ceramide profile in large cohorts of animals. Consequently, additional research should focus on ceramide panel combinations, perhaps with other lipids or cytokines. It has been found, for instance, that circulating fatty acylcarnitine levels are favorably correlated with FA, ceramide, and dihydro-sphingomyelin levels (121). The accumulation of fatty acylcarnitines and ceramides is facilitated by excess FA and defective FA oxidation (122). Although circulating fatty acylcarnitines increased in all cows throughout the peripartum period, increasing prepartum obesity was associated with a more considerable increase in plasma FA and fatty acylcarnitines (121). Since ceramides can be generated from different cellular pathways and dihydro-sphingomyelin is generated from *de novo* dihydro-ceramides, dihydro-ceramides could be used as a proxy for monitoring *de novo* ceramide synthesis. The concurrent increases in ceramide and dihydro-sphingomyelin concentrations indicate that excess FA that is not oxidized is diverted to *de novo* ceramide production and then released into the circulation, where it may inhibit the action of insulin on target tissues. Therefore, the ceramide panel combinations with fatty acylcarnitines and/or dihydro-sphingomyelin could be potential biomarkers.

In addition, there are two aspects to consider when implementing ceramides as biomarkers in practice. First, the stability, extraction procedures, and quantification of ceramides (through mass spectrometry, for example) must be appropriately established. The internal standard must be a stable-isotope-labeled version of the target analyte. Nonetheless, some published investigations employed C17-moieties as standards for all ceramide species, which calls into doubt the reproducibility of the results. Different detection techniques and preanalytical circumstances help explain the contradictory results found in the scientific literature. Second, using ceramides as biomarkers requires specific cutoff values, not only for comparison with healthy controls but also with conditions with which they are associated (e.g., FLD compared with ketosis and retained placenta compared with displaced abomasum). Additionally, ceramides are expected to provide distinct advantages over currently used biomarkers (e.g., NEFA and BHBA) regarding convenience, cost, and disease prediction accuracy.

## 7 Can adiponectin or FGF21 alter ceramides of dairy cows?

In addition to storing energy, adipose tissue synthesizes and secretes several metabolically active hormones, proteins, and other compounds, collectively referred to as adipokines (123). Adipose tissue produces and secretes numerous adipokines, including leptin, adiponectin, chemerin, visfatin, pigment epithelium-derived factor, resistin, fibroblast growth factor 21 (FGF21), retinol-binding protein 4, apelin, omentin, and many others (123).

Adiponectin, predominantly derived from adipocytes, is synthesized as a monomer; however, polymerization of adiponectin is essential to regulate biological activity (124). In dairy cows, adiponectin circulates mainly as a high molecular weight isoform, regardless of the lactation stage (125, 126). In humans, circulating adiponectin with high molecular weight is negatively associated with plasma glucose, insulin, triglycerides, and high-density lipoprotein cholesterol (127). In addition, adiponectin inhibits lipolysis in adipose tissue and lowers insulin resistance by increasing FA oxidation and decreasing triglyceride levels in muscle and liver (127). Adiponectin exerts its effects via two receptors, adiponectin receptor 1 (AdipoR1) and adiponectin receptor 2 (AdipoR2), which are unevenly distributed in different organs (127, 128). Taking note of the opposing effects of adiponectin and ceramides, Holland and Scherer (13) demonstrated that adiponectin receptors (AdipoR1 and AdipoR2) and members of the progesterone and AdipoQ receptor families share a high degree of homology with intracellular ceramide synthases. Later, Vasiliauskaite-Brooks et al. (129) determined the crystal structure of the receptor, which revealed a striking similarity to ceramidases. In addition, they demonstrated that the isolated receptor possesses ceramidase activity (129). These results demonstrate conclusively that adiponectin inhibits ceramide production when NEFA is required for energy production. In insulin-resistant individuals, serum and tissue ceramides are negatively correlated with adiponectin levels (130, 131).

The drop in circulating adiponectin was evident 2 weeks before calving in most trials (125, 132). During the first 3-4 weeks of lactation, adiponectin levels comparable to those approximately 3 weeks before calving were reached or exceeded, having reached a minimum around parturition (125). In contrast, ceramides and glycosylated ceramides accrue throughout the transition from gestation to lactation in overweight cows, while plasma SM, a potential source of ceramides, reaches a minimum at parturition (8). De Koster et al. (133) reported that circulating adiponectin was favorably correlated with insulin sensitivity and inversely correlated with BCS, with adiponectin levels dropping near the end of the dry period. Additionally, in calf adipocytes, TNF-α was found to diminish the transcriptional activity of PPARγ, leading to a decrease in adiponectin production (134). Elevated TNF-α levels within adipose tissue locally could potentially contribute to the reduction of circulating adiponectin in periparturient dairy cows (134). Considering the relationship between ceramides and TNF-α, along with the role of adiponectin receptors in ceramide metabolism, we hypothesize an association between circulating adiponectin and ceramides in periparturient dairy cows, warranting further investigation for validation.

As a member of the endocrine FGF subfamily, FGF21 has gained increased interest for its functional similarities with adiponectin to decrease plasma glucose, circulating lipid levels, and body weight by ameliorating adiposity (135, 136). FGF21 is also regarded as an adipokine (137), and the adipokine adiponectin mediates the metabolic effects of FGF21 on energy metabolism, glucose homeostasis, and insulin sensitivity in the liver and skeletal muscle (138, 139). By attenuating the hepatic influx of adipose tissue-derived FA, Caixeta et al. (140) demonstrated FGF21’s capacity to minimize liver lipid deposition in early-lactating dairy cows. However, Krumm et al. (141) found that FGF21 treatment did not impact plasma glucose or insulin levels, suggesting that FGF21 fails to function as an insulin sensitizer during early lactation. A recent study showed that chronic FGF21 treatment increased glucose disposal with a glucose tolerance test in non-lactating ewes (142). The difference in results between early-lactation cows and non-lactating ewes indicates that insulin-sensitizing effects of FGF21 are conserved in ruminants, increasing the possibility that lactation is an FGF21-resistant condition (142).

**Figure 5:**
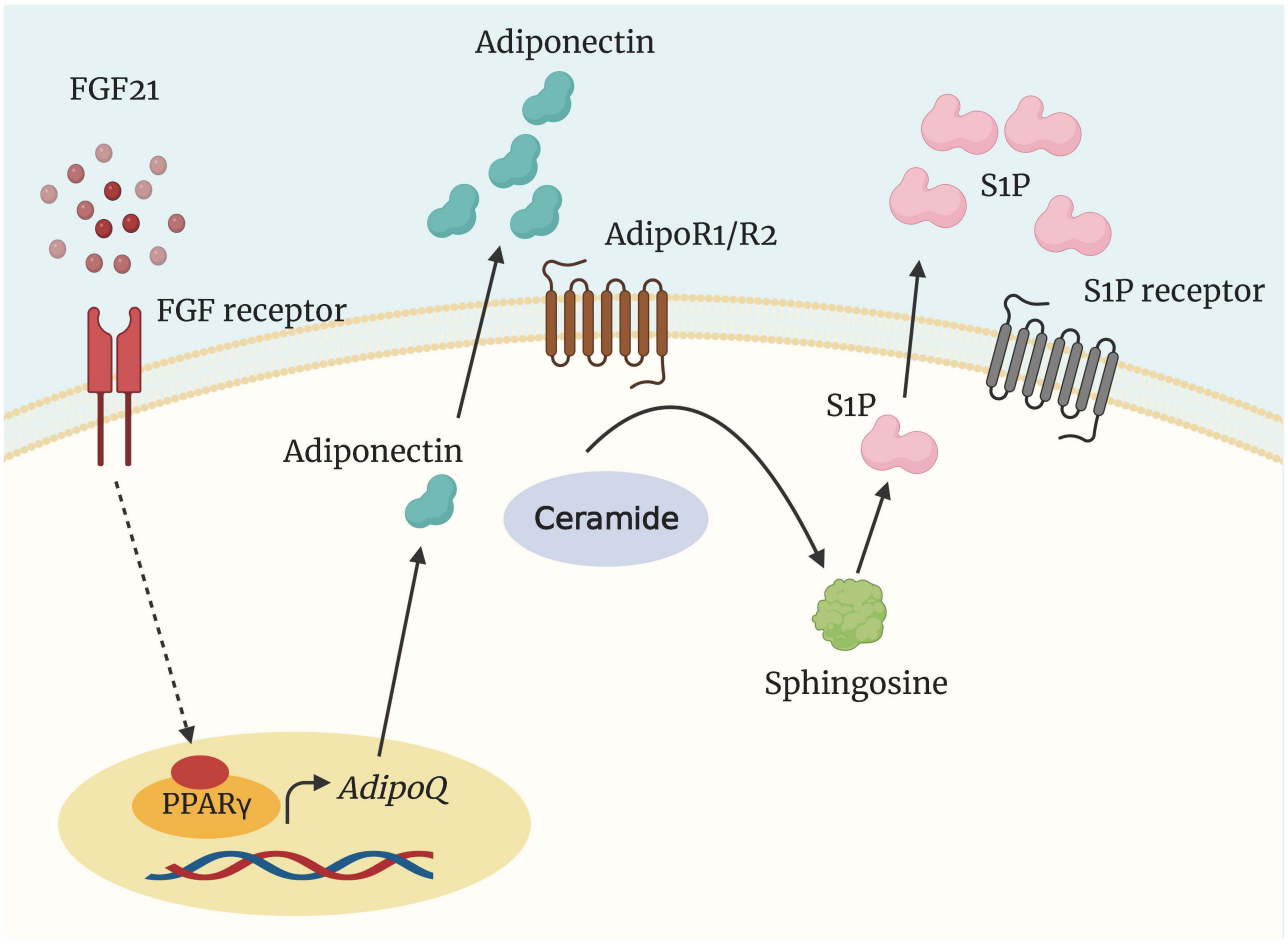
Regulation of ceramide degradation by FGF21 and adiponectin. The insulin-sensitizing and metabolically protective agents FGF21 and adiponectin elicit at least some of their functions through the activation of a ceramidase to generate sphingosine and S1P. AdipoQ, adiponectin gene; AdipoR1/2, adiponectin receptor; FGF21, fibroblast growth factor 21; PPARγ, peroxisome proliferator-activated receptor γ; S1P, sphingosine 1 phosphate.

Holland et al. (138) reported the existence of an FGF21-adiponectin-ceramide axis that controls energy expenditure and insulin signaling. FGF21 was shown to decrease ceramide concentrations while simultaneously increasing adiponectin production selectively. Adiponectin deficiency rendered mice resistant to FGF21-mediated stimulation of metabolism and ceramide reduction (138) (**Figure 5**). Despite not measuring plasma and tissue ceramides, chronic FGF21 infusion did not affect indices of adiponectin production in lactating dairy cows, including plasma adiponectin and adiponectin mRNA abundance (141). In contrast, Lamb et al. (142) observed that FGF21 administration increased plasma adiponectin but did not affect plasma C16:0- and C24:0-ceramide and total ceramides in nonlactating ewes. Therefore, whether the FGF21-adiponectin-ceramide axis is present in early-lactation dairy cows experiencing NEB remains a matter for further research.

## 8 Possible options to lower ceramide levels

Studies in rodents have shown that pharmacologically reducing ceramides prevents or even suppresses the progression of metabolic diseases (23, 143). Although current studies have not been able to confirm a role for ceramides in the development of disease in transition dairy cows, interfering with ceramide metabolism may provide an opportunity to improve cow health, especially in overconditioned cows. Rates of ceramide synthesis are determined mainly by the availability of long-chain saturated FA, which are involved in the initial, rate-limiting step of *de novo* ceramide synthesis (19). SPT explicitly catalyzes the condensation of palmitoyl-CoA and serine to create 3-oxosphinganine in this reaction. Several animal models have demonstrated that the potent SPT inhibitor myriocin, frequently used to suppress *de novo* ceramide biosynthesis, improves insulin sensitivity and hepatic steatosis, and protects against atherosclerosis and cardiomyopathy (127, 144). Other preclinically tested ceramide *de novo* synthesis inhibitors include Fumonisin B1, Fingolimod, and Fenretinide (73). To date, these medication candidates for treating FLD in humans have yet to reach the clinical trial stage. Although myriocin was also shown to inhibit ceramide *de novo* biosynthesis in the liver of ewes (67) and bovine adipocytes (17), myriocin is unsuitable for dairy cows due to its high cost. The administration of myriocin at a dose of 1 mg/kg BW every other day for 17 d, suppresses appetite in ruminants (67). It should be noted that directly inhibiting *de novo* ceramide synthesis may be lethal. Perhaps, we can consider regulating ceramide synthesis from other metabolic pathways, such as sphingomyelin hydrolysis and the salvage pathway.

The latest scientific literature describes a strong relationship between dietary phytochemicals and sphingolipid-mediated ceramide regulatory pathways (145). Diverse phytochemicals with therapeutic qualities, such as antitumor, anti-inflammatory, anti-diabetic, and cardioprotective activities, may control ceramides in rodent models. For example, anthocyanins have been found to improve insulin resistance, normalize insulin signaling, reduce body weight, and correct abnormal liver function by reducing circulating ceramides (146). Resveratrol has also been reported for its ability to restore insulin signaling and prevent palmitate-induced ceramide formation while partially generating dihydroceramides (147). However, very few researchers have investigated the impact of phytochemicals on ceramide levels in dairy cattle. Our latest study showed that feeding citrus flavonoids decreased fecal GlcCer(d18:1/20:0), Cer(d18:0/24:0), and Cer(d18:0/22:0) in mid-lactation dairy cows (148). In dairy cows fed a high-starch diet, it was observed that supplementing citrus flavonoids decreased plasma concentrations of Cer(d18:1/18:0), and Cer(d18:0/24:0) (149). Grape seed and grape marc meal extract (GSGME) supplementation during the periparturient period decreased the liver expression of a substantial number of genes implicated in the ER stress-induced unfolded protein response and inflammatory processes in dairy cows (150). Lipidomic research has demonstrated that polyphenols from GSGME do not affect plasma ceramide concentrations or molecular profiles (150). Thus, the beneficial effects of GSGME on liver inflammation and ER stress are independent of ceramide metabolism. It is important to remember that structural differences among phytochemicals make them effective under different situations and varying degrees. The potential of phytochemicals to influence ceramide metabolism in transition dairy cows warrants further investigation.

## 9 Conclusions and perspectives

Countless advances in understanding the transition to the lactation period, nutrient partitioning, and tissue level regulation of metabolism have been made over the last two decades; however, the unanswered questions and remaining opportunities still need to be addressed. New molecular mechanisms emerge as targets for the prevention and treatment of NEB-related metabolic disorders, such as FLD and ketosis, which are the subject of ongoing research. In earlier research, lipidomic profiling has yielded fresh insights into the molecular pathways behind liver and adipose tissue metabolic dysfunctions in transition dairy cows. There is increasing evidence that the major sphingolipids, ceramides, may be involved in hepatic and adipose tissue disorders.

It is becoming increasingly apparent that the severity of changes in these simple metabolites cannot explain periparturient diseases and disorders. Caution should be exercised since interpreting biomarkers as causal agents of metabolic disorders is not in accordance with the purpose of epidemiological studies. It is still unclear whether ceramide regulation in early lactation diseases occurs as a response to pathogenic causes, such as metabolic stress or inflammation, or whether ceramides actively contribute to disease development. That is, whether ceramide plays a role as a passenger or driver in extrahepatic insulin resistance and inflammation and oxidative stress in liver and adipose tissue in transition dairy cows remains unknown. This question requires further investigation using dairy cows. If the significance of ceramides as a contributor to metabolic disorders is confirmed, these findings may have diagnostic and therapeutic implications for metabolic diseases. In addition to evaluating the efficacy of circulating ceramide species as diagnostic predictors of metabolic disorder risk relative to the regularly used biomarkers NEFA and BHBA, scientists should also assess the diagnostic value of plasma ceramide species.

Furthermore, several pertinent questions remain to be addressed. Within the scope of this review, many knowledge gaps remain. First, the diversity of the bioactive sphingolipid network in dairy cows makes it highly challenging to investigate the ceramide pool, especially in the context of the complete body, where unique activities of distinct tissues and organs interact and converge. Equally essential is the need to comprehend the mechanisms of action of the many ceramide species. Fundamental discovery biology exploring the role of these lipids in cellular activity is of paramount significance for creating new therapeutics and comprehending the etiology of dairy cow metabolic problems.

Second, what genetic factors determine ceramide accumulation in dairy cows with NEB? Multiple single-nucleotide polymorphisms have been discovered in human ceramide-modifying enzymes. However, ceramide’s molecular genetic basis remains mostly unexplained. Will research demonstrate that these potential mutations have a functional impact on sphingolipid profiles or metabolic disease risk? If true, can these data be utilized to establish breeding techniques to reduce ceramides and improve cows’ health? Expanding and refining our understanding of the genetic regulatory network of ceramide metabolism could lead to developing intervention strategies for preventing and/or treating metabolic problems in dairy cows.

Third, is it possible to develop novel strategies for lowering ceramide levels, modifying insulin sensitivity, and improving cow health? Given the recent resonance of ceramide in transition dairy cows’ health, they merit investigation as potential new therapeutic targets in metabolic diseases. However, a direct application of ceramide inhibitors based on rodent trials seemed impossible in dairy farms. We expect that larger-scale trials of dairy cows will reveal insights into the entire complicated ceramide system, leading to new directions in transition cow nutrition and management.

## Author contributions

YZ: Conceptualization, Funding acquisition, Writing – original draft. HZ: Software, Visualization, Writing – original draft. LL: Investigation, Software, Writing – original draft. SY: Software, Writing – original draft. ML: Writing – review & editing. LJ: Conceptualization, Writing – review & editing.
